# Assessing the Impact of Agricultural Information Utilization on Wheat Productivity of Smallholder Farmers: Evidence From Northwest Ethiopia

**DOI:** 10.1155/tswj/7605699

**Published:** 2025-09-30

**Authors:** Zebenay Shitaye, Bamlaku Tadesse, Koyachew Enkuahone

**Affiliations:** ^1^College of Agriculture & Environmental Science, Bahir Dar University, Bahir Dar, Ethiopia; ^2^College of Agriculture & Natural Resource, Debre Markos University, Debre Markos, Ethiopia; ^3^Institute for Peace and Security Studies (IPSS), Addis Ababa University, Addis Ababa, Ethiopia

**Keywords:** impact, information utilization, productivity, PSM, smallholder farmer

## Abstract

Smallholder farmers in Ethiopia face significant challenges in accessing reliable agricultural information due to limited extension services, inadequate infrastructure, low literacy levels, and restricted access to digital tools. These barriers negatively impact farm productivity. This study investigates the effect of agricultural information utilization on wheat productivity in the East Gojjam Zone of the Amhara Region. Data were collected through a cross-sectional survey of 403 wheat-producing households. To address potential selection bias, a propensity score matching (PSM) model was applied, incorporating variables such as farmer mobility, access to extension services, availability of farm inputs, proximity to markets, and exposure to electronic media. The findings reveal that farmers who accessed and effectively used agricultural information achieved an average wheat yield increase of 19.675% per hectare compared to nonusers. This result underscores the crucial role of information access in improving productivity, particularly when supported by better mobility, strong extension services, reliable input supply, market accessibility, and media connectivity. Based on these insights, the study recommends strengthening the agricultural extension system, improving input supply chains, enhancing rural market infrastructure, expanding access to electronic media, and promoting cooperative-based information dissemination as key strategies to support sustainable agricultural development in Ethiopia.

## 1. Introduction

Agriculture remains a cornerstone of economic development in many less developed countries (LDCs), especially in sub-Saharan Africa, where it is seen as a pathway to poverty alleviation and inclusive growth [1, 2]. In Ethiopia, the sector contributes approximately 32.1% to the GDP, employs about 85% of the labor force, generates over 90% of export earnings, and supplies 70% of the raw materials for agroindustries [3]. Despite its vital role, Ethiopian agriculture faces persistent structural and institutional challenges: small landholdings, erratic rainfall intensified by climate change, declining soil fertility, limited market integration, and low adoption of modern technologies [4–6].

A major constraint exacerbating these challenges is inadequate access to reliable agricultural information. In the digital age, information and communication technologies (ICTs) are increasingly recognized as enablers of transformation in agriculture, facilitating improved access to timely, relevant, and actionable information [1, 7]. ICTs ranging from mobile phones and apps to radio and internet platforms empower farmers to make informed decisions about inputs, practices, and market opportunities [8, 9]. Consequently, agricultural extension systems must embrace not only traditional outreach methods but also digital innovation to ensure interactive and inclusive communication among stakeholders [10, 11].

Access to agricultural information reduces uncertainty and risk associated with production and marketing, thereby increasing efficiency and farm incomes [12, 13]. Farmers who receive timely data on weather forecasts, pest outbreaks, crop management techniques, and market prices are more likely to adopt innovations, increase yields, and enhance food security productivity [14, 15]. However, in rural Ethiopia, barriers such as limited infrastructure, low digital literacy, language diversity, and uneven extension coverage constrain access to and use of such information [16]. Although mobile-based services and farmer cooperatives are making progress in knowledge dissemination, their reach and effectiveness remain inconsistent [17].

Moreover, many rural communities are not fully aware of their information gaps or the tools available to bridge them [18]. While previous studies have examined factors influencing access to agricultural information, theoretical frameworks often lack a holistic view, failing to consider the interplay of sociocultural, institutional, and technological dimensions [19, 20]. Additionally, the rural–urban digital divide remains underexplored in Ethiopia's agricultural policy discourse [21]. On the practical side, the implementation of ICT-based interventions often lacks scalability, coordination, and long-term impact evaluation [2, 16].

At the microlevel, understanding farmers' socioeconomic profiles such as age, education, gender, farm size, and access to infrastructure is essential to tailor interventions effectively. At the macrolevel, misaligned policies that ignore these dynamics risk inefficiency and stagnation in agricultural growth. Therefore, rigorous empirical analysis is needed to assess how access to and utilization of agricultural information influences productivity outcomes. Additionally, examining the impact of agricultural information utilization on productivity is crucial for enhancing overall agricultural output and food security. This study seeks to bridge these theoretical and practical gaps by pursuing the following objectives:

• *To analyze the socioeconomic characteristics of smallholder farmers* in the East Gojjam Zone of Ethiopia.
• *To examine the effect of agricultural information utilization on agricultural productivity* among wheat-producing households.

## 2. Review Literature

A growing body of literature highlights the diverse factors influencing smallholder farmers' access to and utilization of agricultural information, which significantly impacts farm productivity. Numerous studies identify key socioeconomic characteristics such as age, education, gender, household size, income, farming experience, and farm size as critical determinants of how and to what extent farmers use agricultural information. For instance, [22] found that income level, education, age, perception, and access to training are major determinants of ICT tool adoption for agricultural information among Nigerian farmers. Similarly, research on sorghum producers confirms that age, education, and landholding size play a significant role in shaping how farmers engage with agricultural knowledge systems.

Age is often linked to accumulated farming knowledge, which may enhance a farmer's ability to interpret and utilize agricultural information effectively. However, younger farmers tend to adopt new technologies more readily, especially when digital tools are involved. Education is another strong predictor of information utilization. Literate farmers are better equipped to process complex information, make informed decisions, and adapt to modern technologies. Larger landholders are generally more commercially oriented and tend to pursue agricultural information more actively, partly because they are better able to manage risks and invest in improved technologies [23].

Institutional factors also play a crucial role in supporting agricultural information utilization. Key institutional mechanisms include extension services, cooperatives, access to credit, and the availability of ICT infrastructure. Public and NGO-based extension services have historically played a central role in disseminating agricultural knowledge; however, disparities in coverage and quality remain major challenges in countries like Ethiopia [16, 24]. Local organizations and farmer cooperatives contribute to peer learning and provide platforms for shared knowledge, which often boosts adoption rates through social learning [17]. Access to credit is another essential institutional factor, as farmers with financial support are more likely to afford inputs and invest in productivity-enhancing information and technologies [25].

ICTs are increasingly recognized as transformative tools in agricultural development. Mobile phones, radio programs, internet services, and mobile apps offer new pathways for delivering timely and localized information on weather, prices, pests, and farming practices. Nonetheless, these ICT-based interventions are often limited by infrastructural deficits, low digital literacy, gender disparities, and language barriers [2, 26]. Global experiences affirm the impact of ICTs in agriculture. In Kenya, mobile platforms like *iCow* have improved farmer decision-making and productivity [27]. In India, the *e-Choupal* initiative has revolutionized rural agriculture by connecting farmers to markets and best practices, resulting in increased efficiency and income [28]. In Nigeria, the integration of radio and mobile communication has significantly improved the accessibility and use of agricultural innovations [8].

The relationship between agricultural information and productivity is well established. Access to actionable and reliable information helps farmers manage risks, optimize input use, and improve decision-making, all of which contribute to increased yields and income. Lv et al. [13], for example, demonstrated in China that agricultural information significantly improved net income among smallholder farmers. Similarly, [29] found that Tanzanian farmers who relied on ICT-enabled services achieved higher productivity than those using traditional communication channels.

Despite the progress, several theoretical and practical gaps remain in the literature. Many studies focus predominantly on access to agricultural information, with limited attention to actual utilization behavior and its drivers. There is also a lack of holistic approaches that integrate behavioral, institutional, and contextual dimensions. Moreover, the impact and scalability of digital interventions often lack rigorous evaluation. In Ethiopia specifically, the rural–urban digital divide, along with gender and youth disparities in digital tool adoption, is insufficiently explored [20, 21]. These limitations point to the need for more context-sensitive and evidence-based strategies to promote effective information utilization.

**Figure 1:**
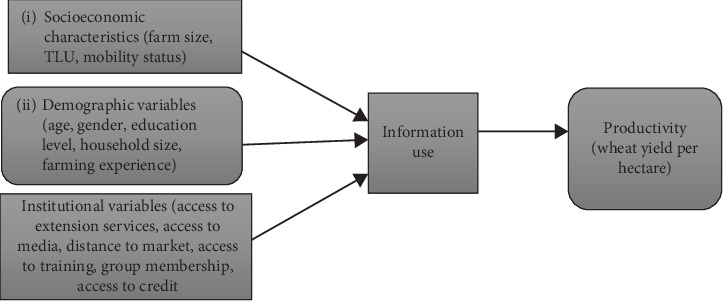
Conceptual framework of the study.

In summary, both individual (age, education, gender, experience, and farm size) and institutional (extension services, cooperatives, credit access, and ICT infrastructure) factors critically shape how smallholder farmers access and use agricultural information. While the link between information and productivity is evident, the extent and effectiveness of utilization vary widely depending on context. This underscores the importance of integrating socioeconomic, institutional, and technological perspectives in agricultural development policies. To guide this study, a conceptual framework rooted in adoption–diffusion theory and the access–use–impact model has been proposed. This framework links farmer characteristics and institutional factors to agricultural information utilization and, ultimately, productivity outcomes (Figure 1).

## 3. Research Methodology

### 3.1. Study Site Description

This study was conducted in East Gojjam Zone, located in the Amhara National Regional State of Ethiopia. The zone features diverse topography ranging from 500 to 4154 m above sea level, contributing to varied agroecological conditions critical for agriculture [30]. East Gojjam is bordered by the Oromia Region to the south, West Gojjam to the west, South Gondar to the north, and South Wollo to the east. It comprises 18 districts and has a population of approximately 2.5 million across 14,004.47 km^2^, with a population density of about 170 people/km^2^ [31].

The study focused on three districts: Gozamin, Debre Elias, and Baso Liben. Gozamin covers 121,781 hectares, of which 50,084 hectares are used for agriculture. It features mostly Nitisol soils and Weyna Dega agroecology, with rainfall between 1448 and 1808 mm and temperatures ranging from 11°C to 25°C [32]. Debre Elias, with elevations between 800 and 2200 m, is suited for wheat, maize, and teff production. The district receives about 1150 mm of rainfall annually, and 98% of its area falls within the Woina Dega zone [33]. Baso Liben is known for cereal surplus production, particularly wheat and teff, supported by rainfall ranging from 1200 to 1600 mm and elevations between 1500 and 2500 m [34, 35]. Despite minimal industrial development, agriculture remains the dominant economic activity in all three districts.

### 3.2. Method of Sampling Procedure and Methods of Data Collection

#### 3.2.1. Research Design

The study employed a cross-sectional research design, enabling the collection of data at a single point in time from a range of participants. This design is suitable for analyzing relationships between agricultural information utilization and productivity among smallholder farmers [36].

#### 3.2.2. Method of Sampling Technique

A multistage sampling technique was used to ensure representativeness. In the first stage, East Gojjam Zone was purposively selected due to its agricultural importance and diversity. In the second stage, three districts were chosen based on their agricultural activity and accessibility. From each district, two kebeles (the smallest administrative units in Ethiopia) were randomly selected, resulting in a total of six kebeles. In the third stage, individual farmers were selected using a computer-generated random sampling method from each kebele's household list, ensuring that each farmer had an equal chance of selection and minimizing sampling bias.

#### 3.2.3. Data Sources and Data Types

The study utilized both primary and secondary data sources to ensure a comprehensive analysis. Primary data sources included structured surveys administered to farmers, semistructured interviews with agricultural extension workers, and focus group discussions (FGDs) among farmer groups. These methods provided quantitative data on socioeconomic characteristics as well as qualitative insights into experiences and perceptions regarding agricultural information utilization. Secondary data sources consisted of government reports, agricultural databases, and relevant literature, which helped to contextualize the findings. The data types encompassed quantitative data, such as age, income, and farm size, for statistical analysis, along with qualitative data from interviews and discussions that identified themes and patterns. This mixed methods approach enhanced the study's robustness and depth.

#### 3.2.4. Sample Size Determination

To determine the sample size for the study, three key criteria were considered: the level of precision (sampling error), the level of confidence, and the degree of variability among the population's measured attributes. The Cochran (1963) formula was applied to calculate the sample size:

 $$\begin{array}{c} n=\frac{z^{2}.p.q}{{\left(e\right)}^{2}} \end{array}$$

where *Z* is the *z* value for a 95% confidence level (1.96), *p* = 0.5 (assumed proportion), *q* = 1 − *p* = 0.5, and *e* = 0.05 (the desired level of precision). Substituting the values,

$$\begin{array}{cc} \; & n=\frac{{\left(1.96\right)}^{2}.\left(0.5\right).\left(0.5\right)}{{\left(0.05\right)}^{2}},\\ n=\frac{0.9604}{0.0025}\approx 384+5\%\text{of }384=403. \end{array}$$

To account for nonresponses, a 5% buffer was added, resulting in a final sample size of approximately 403 farmers [37].

#### 3.2.5. Methods of Data Collection

This study used both quantitative and qualitative methods to ensure a comprehensive understanding of agricultural information utilization. Quantitative data were collected through structured questionnaires administered to 403 randomly selected smallholder wheat farmers. The questionnaire included closed-ended questions covering socioeconomic characteristics, farming practices, access to agricultural information, and productivity. It was pretested and conducted through face-to-face interviews by trained enumerators.

Qualitative data were gathered through semistructured interviews with agricultural extension workers, local leaders, and development agents. Additionally, FGDs were conducted with farmers (8–10 participants per group) to explore shared experiences and challenges.

Relevant secondary data from government reports, research centers, and existing literature were also used to support and validate the findings. This mixed methods approach allowed for both statistical analysis and in-depth insights, ensuring a well-rounded understanding of the research problem.

### 3.3. Data Analysis

The study used a mixed methods approach, combining quantitative and qualitative analyses. Quantitative data were analyzed using descriptive statistics and propensity score matching (PSM) to estimate the causal impact of agricultural information utilization on wheat productivity. PSM was employed to control for selection bias by matching users and nonusers of information based on similar observable characteristics such as age, education, farm size, and access to services. The analysis, conducted in Stata 17 using the psmatch2 command, applied nearest neighbor, kernel, stratification, and radius matching techniques. The average treatment effect on the treated (ATT) was calculated, and balance diagnostics confirmed effective matching. Qualitative data from interviews and FGDs were analyzed thematically to complement and enrich the quantitative findings.

#### 3.3.1. Econometric Model Specification

PSM was selected for this study due to its effectiveness in addressing selection bias and estimating causal effects in observational data. PSM controls for confounding variables by matching treated and untreated farmers with similar propensity scores, thus reducing bias in estimating the impact of agricultural information utilization on productivity. This method enhances the robustness of estimates and facilitates causal inference by creating balanced comparison groups, which are crucial in agricultural research where various external factors can influence outcomes. Additionally, PSM's flexibility allows it to adapt to high-dimensional data, making it a suitable approach for analyzing the diverse characteristics of farmers in the study area.

Introduced by [38], PSM effectively estimates mean impacts without arbitrary assumptions about functional forms or error distributions. This study is aimed at assessing the impact of agricultural information technology on wheat yield and income among small farmers by matching participating farmers with similar nonparticipants based on preintervention characteristics, thereby reducing bias from confounding variables [39].

The final analysis calculates the mean difference in wheat yield between the two groups, approximating the average treatment effect (ATE). By employing logistic regression to determine propensity scores, PSM enhances the validity of benefit estimates related to technological adoption in agriculture, addressing the challenge of estimating impacts in observational studies [38]. Overall, PSM addresses the challenge of estimating impacts in observational studies where individual outcomes under both treatment conditions cannot be observed simultaneously.

Let *Yi*^*T*^ and *Yi*^*C*^ represent the outcomes for users and nonusers, respectively. The difference in outcomes between treated and control groups is expressed as follows:

 $$\begin{array}{c} \Delta_{i}=Y_{i}^{T}-Y_{i}^{C} \end{array}$$

where

• *Y*_i_^T^ is the wheat yield (in quintals per hectare) for the *i*-th household when using agricultural information.
• *Y*^iC^ is the yield for the *i*-th household when not using this technology.
• Δ_*i*_ indicates the change in outcome due to technology use.

This can be reformulated in causal effect notation, defining *D*_i_ = 1 for treated individuals and *D*_i_ = 0 for nontreated. The ATE is expressed as follows:

 $$\begin{array}{c} ATE=E\left(Y_{i}^{T}\left|D_{i}=1\right.\right)-E\left(Y_{i}^{C}\left|D_{i}=0\right.\right) \end{array}$$

where

• *E*(*Y*_*i*_^*T*^|*D*_*i*_ = 1) is the expected outcome for treated individuals.
• (*D*_*i*_ = 1).*E*(*Y*_*i*_^*C*^|*D*_*i*_ = 0) is the expected outcome for nontreated individuals.

The ATT is given by

 $$\begin{array}{c} ATT=E\left(Y_{i}^{T}-Y_{i}^{C}\left|D_{i}=1\right.\right)=E\left(Y_{i}^{T}\left|D_{i}=1\right.\right)-E\left(Y_{i}^{C}\left|D_{i}=1\right.\right). \end{array}$$

A key challenge in impact estimation is the inability to observe individual outcomes under both treatment conditions simultaneously, leading to potential biases in estimating the ATE [40]. To address this, researchers construct counterfactual outcomes through matching, relying on two main assumptions.

The conditional independence assumption (CIA) states that treatment assignment is independent of outcomes when conditioned on observed characteristics:

 $$\begin{array}{c} Y_{i}T-Y_{i}C⊥\left(D\left|X\right.\right). \end{array}$$

The CIA posits that participation in a program is random among similar individuals, helping to avoid self-selection bias [41]. However, with many conditioning variables, finding identical households becomes difficult.

To facilitate this, [38] introduced the “propensity score,” which is the probability of receiving treatment given characteristics *X*:

 $$\begin{array}{c} P\left(X_{i}\right)=\text{Pr}\left(D_{i}=1\left|X_{i}\right.\right). \end{array}$$

Here, *D*_i_ = 1 indicates treatment. When estimating propensity scores, all relevant variables affecting both treatment utilization and outcomes are included. The ATT is calculated as follows:

 $$\begin{array}{c} ATT=E\left(Y_{i}T\left|P\left(X\right),D_{i}=1\right.\right)-E\left(Y_{i}C\left|P\left(X\right),D_{i}=1\right.\right). \end{array}$$

The common support assumption ensures that the propensity score lies between 0 and 1:

 $$\begin{array}{c} 0<P\left(X\right)<1. \end{array}$$

This allows for effective matching of treated and control groups [42]. Together, these assumptions form the strong ignorability assumption [38] describing assumption one and two together as the strong ignorability assumption.

Caliendo and Kopeinig [40] outline the main steps in PSM: estimation of the propensity score, choice of a matching algorithm, verification of common support, and checks of matching quality. In this study, a logistic regression model is employed, using agricultural information as the dependent variable and socioeconomic factors as explanatory variables to assess the adoption of agricultural technologies

## 4. Results, Discussion, and Recommendation

### 4.1. Descriptive Summary of Selected Variables Used in Estimations

The analysis in Table 1 highlights key differences between users and nonusers of improved agricultural information, focusing on several important socioeconomic and institutional variables. Farmer mobility is significantly higher among users, indicating that these farmers are more able to travel to markets, training centers, and service providers. This greater physical access enhances their exposure to new information, technologies, and opportunities. The practical implication is that improving rural transport infrastructure, such as roads and public transportation systems, can boost farmers' mobility and in turn increase their access to essential agricultural services.

Access to extension services is also notably higher among users. These farmers benefit from more frequent visits by extension agents and better access to technical advice, which helps them apply improved farming practices. This implies that expanding the reach and capacity of extension programs, especially in remote or underserved areas, can significantly improve farm productivity and promote technology adoption.

The use of printed information outlets, including brochures, manuals, and posters, is more common among users, reflecting greater engagement in self-directed learning. These materials provide essential information on planting techniques, pest control, and postharvest handling. The practical implication here is the importance of producing and distributing user-friendly, localized print materials, which can serve as effective communication tools for both literate and semiliterate farmers. In addition, access to farm inputs, such as improved seeds, fertilizers, and pesticides, is higher among users. Reliable access to these inputs enables farmers to enhance their yields and improve the quality of their produce. This suggests the need to integrate information dissemination with input supply systems such as through cooperatives, agrodealers, or public–private partnerships to ensure that farmers not only know what to use but can also access it.

Utilization of electronic media sources, including radio, television, and mobile internet, is more frequent among users, indicating their greater reliance on diverse and timely sources of agricultural information. These channels are crucial for receiving weather updates, market prices, and farming tips. The practical implication is that expanding digital infrastructure and promoting media literacy among rural farmers can help bridge the information gap and support real-time decision-making.

Finally, distance from home to market centers is generally shorter for users, allowing them easier and more regular access to input suppliers and buyers. Proximity to markets reduces transaction costs and increases the likelihood of selling produce at favorable prices. This underlines the need for rural development strategies that prioritize road construction, market establishment, and transport services to improve connectivity and economic participation.

Overall, the observed differences in these variables demonstrate how access to improved agricultural information contributes to better resource utilization, informed decision-making, and improved livelihoods. These findings reinforce prior research, such as [43] which emphasizes the transformative role of information accessibility in enhancing smallholder productivity and resilience.

### 4.2. Agricultural Information Utilization by Gender

**Figure 2:**
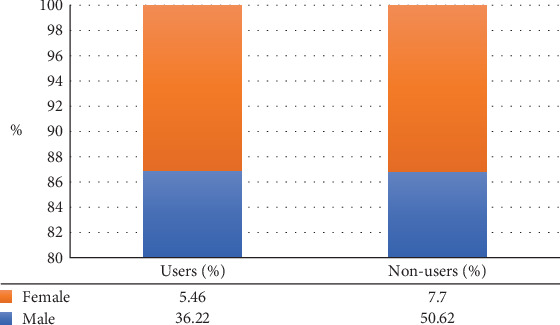
Agricultural information utilization by gender. Source: Filed data, 2023.

The findings presented in Figure 2 reveal a significant gender disparity in the utilization of agricultural information among smallholder farmers. While 50.62% of male respondents were nonusers, only 7.7% of female respondents fell into this category. Similarly, 36.22% of males were identified as users compared to just 5.46% of females. These figures highlight the considerable exclusion of women from formal agricultural information systems, which has serious implications for gender equity and farm productivity.

This disparity is deeply rooted in structural and sociocultural constraints that govern gender roles in many rural Ethiopian communities and across sub-Saharan Africa more broadly. Agriculture is often perceived as a male domain, while women's contributions, though substantial, are typically confined to subsistence production or unrecognized labor [44]. As a result, women have less access to formal channels of information, which are essential for adopting improved farming technologies and practices.

One of the primary constraints is limited mobility and access to agricultural services. Women frequently bear the burden of household responsibilities, including childcare, food preparation, and water collection, leaving them with less time to attend training sessions or visit extension centers [45]. In addition, extension services are often male-dominated and biased toward working with male heads of households [46], further marginalizing women and reinforcing gender stereotypes within agricultural systems.

Moreover, women's reliance on informal networks such as family, neighbors, and local groups for agricultural information reflects a lack of access to structured and reliable sources [47]. Although these informal channels are valuable for sharing experiences, they often lack technical depth or may transmit outdated or incomplete knowledge. In contrast, men are more likely to access formal sources such as extension agents, training workshops, printed materials, or mass media, which provide more comprehensive and updated agricultural information.

Another barrier is the social expectation and power imbalance that discourages women from participating actively in mixed-gender training sessions or voicing their opinions during community meetings [48]. These constraints reduce women's visibility in agricultural development initiatives and limit their ability to act on new knowledge.

These sociocultural limitations not only hinder women's access to agricultural information but also perpetuate a cycle of low productivity and economic vulnerability. Without reliable information, women are less likely to adopt improved technologies, access markets effectively, or make informed decisions about input use [49]. Addressing these barriers requires gender-transformative strategies that go beyond equal distribution of information to tackle the root causes of inequality.

Practical strategies include recruiting more female extension agents [50], delivering women-targeted training programs, using gender-sensitive communication channels (e.g., local radio, group meetings, and mobile SMS), and strengthening women's farmer groups. Institutional reforms must also be made to ensure that extension services recognize and respond to women's needs.

In conclusion, the gender disparity in agricultural information utilization is a reflection of broader structural inequalities that must be addressed to ensure inclusive agricultural growth. Supporting women's access to accurate and timely agricultural information not only is a matter of fairness but is also essential to increasing overall farm productivity, household welfare, and food security [51].

### 4.3. Landholding Size of Respondents

Farm size is a crucial factor influencing the utilization of agricultural information among smallholder farmers in specific regions. According to the findings in Table 2, approximately 15.88% of farmers own less than 1 hectare of land, while the majority (55.33%) own between 1 and 2 hectares. Additionally, 28.77% of respondents possess more than 3 hectares.

The size of landholdings significantly affects farmers' ability to access credit and use their land as collateral. Generally, farmers with smaller plots show less enthusiasm for adopting innovative and advanced technologies compared to those involved in large-scale, commercial farming [23, 52].

This trend can be attributed to several factors: Smaller landholders often have limited resources and may be more risk-averse, which hinders their willingness to invest in new technologies. In contrast, farmers with larger landholdings may have greater financial security and access to information, enabling them to experiment with and adopt new agricultural practices more readily. Thus, addressing the challenges faced by smallholder farmers, particularly those with limited land, is essential for promoting the effective use of agricultural information and enhancing overall productivity.

### 4.4. Factors Influencing Wheat Productivity Information Utilization

This section outlines the estimation process and identifies factors influencing the utilization of agricultural information for wheat productivity. The logit regression results presented in Table 3 reveal that 17 explanatory variables were hypothesized to determine the utilization of agricultural information for wheat productivity. According to the computed coefficients, six explanatory variables significantly affect information utilization at various probability levels.

The analysis reveals that mobility has a statistically significant negative influence on smallholder farmers' utilization of agricultural information, at the 1% significance level. This finding indicates that limited mobility restricts farmers' ability to access timely and relevant information needed to enhance their farming productivity. Reduced mobility often stems from infrastructure-related barriers, such as poor rural road networks, lack of affordable transportation, and long distances to extension offices or market centers. In many rural areas, the absence of reliable infrastructure makes it difficult for farmers especially those in remote locations to physically reach sources of information such as farmer training centers, demonstration plots, cooperatives, or agricultural input suppliers. As [53] suggests, poor infrastructure not only limits market integration but also acts as a constraint on knowledge dissemination and service delivery. Thus, improving rural infrastructure, particularly road access and transport systems, could enhance farmers' mobility and facilitate greater engagement with agricultural information systems, ultimately contributing to improved productivity and livelihoods.

*Access to media*: Both printed and electronic media positively influence smallholder farmers' utilization of agricultural information for their farming productivity at a 1% significance level. This finding is supported by [54], who demonstrated that electronic information sources such as radio, mobile phones, and television are pivotal for farmers in accessing timely and relevant agricultural information.

*Access to extension services*: Access to agricultural extension services positively influences smallholder farmers' utilization of agricultural information for their farming productivity, also at a 1% significance level. This underscores the importance of these services in providing farmers with critical knowledge and support, aligning with studies by [55, 56].

*Access to farm inputs*: The study establishes that access to farm inputs significantly and positively influences the utilization of agricultural information for wheat productivity at a 1% significance level. This highlights the necessity of ensuring that farmers have adequate access to quality inputs, which are vital for effective agricultural practices [57].

*Distance to market centers*: The distance from a farmer's residence to market centers has a significant negative impact on the utilization of agricultural information for wheat productivity at a 5% significance level. This aligns with research from [58, 59], which indicates that greater distances to markets can deter farmers from adopting advanced agricultural technologies and negatively affect their overall income. These quantitative findings were further validated by insights from FGDs and key informant interviews (KIIs), which revealed that farmers in remote areas often face serious mobility and market access challenges that constrain their ability to use agricultural information effectively. Development agents and local extension officers interviewed also stressed that poor road infrastructure, limited farm input supply, and weak media outreach remain critical obstacles, while farmers with better access to services, inputs, and diverse communication channels are more likely to utilize agricultural information and improve their wheat productivity. This combined evidence underscores the complex interplay of factors influencing information utilization and calls for targeted interventions to enhance infrastructure, expand input supply, strengthen extension services, and improve media access to promote sustainable productivity and livelihoods.

### 4.5. Verification of the Balancing Property and Identifying Common Support Region

The matching analysis aimed at reducing bias between the treated and control groups yielded several important findings. First, the postmatch means of the covariates across the two groups were similar, satisfying the balance property, which indicates that the matching process effectively aligned the characteristics of the groups. Statistical testing through **t**-tests revealed no statistically significant differences between the matched treatment and control groups, reinforcing the efficacy of the matching method. Additionally, the values of Rubin's **B** (30.4) and **R** (0.96) served as strong indicators of balance after matching, with only a small amount of bias remaining (Table 4). A substantial reduction in bias was observed, as no important differences in covariates between matched participants and nonparticipants were detected. Furthermore, the pseudo − *R*^2^ in the logit model decreased after matching, and the **p** value from the likelihood ratio test became insignificant, which typically suggests a better fit of the model. Overall, these results indicate that the matching method successfully aligned the treated and control groups, enhancing their comparability for subsequent analyses and improving the reliability of the conclusions drawn from the data.

The common support assessment was a crucial step in ensuring that both users (those who adopted the technology) and nonusers (those who did not) were comparable in terms of their propensity scores. This is essential for avoiding biases in estimating the treatment effect. Among the total of 403 observations analyzed, only seven households from the untreated group (1.73%) were found to fall within the common support region. This low percentage suggests that very few nonusers have similar characteristics to the users, which may limit the generalizability of the findings.

**Figure 3:**
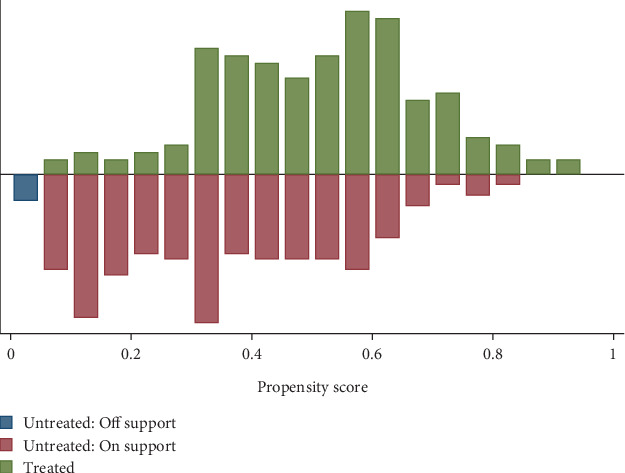
Propensity score graph all (235) untreated and (161) treated on common support region.

However, there was considerable overlap in the propensity scores between the two groups, as illustrated in Figure 3. This overlap indicates that the common support criterion was satisfied, providing a sufficient range of scores for reliable comparisons between users and nonusers. Overall, these steps enhance the robustness of the matching process, enabling a more accurate estimation of the treatment effect on productivity for households utilizing improved agricultural information technologies.

### 4.6. ATT

This section explores the impact of agricultural information utilization on wheat farm productivity at the household level. The analysis reveals a statistically significant increase in productivity for households that have adopted improved agricultural information technologies within the study area.

The data presented in Table 5 indicates that the use of agricultural information positively influences wheat productivity among farmers. Specifically, farmers who utilized agricultural information experienced an average increase of approximately 223 kg in wheat yield compared to those who did not, as determined using the nearest neighbor matching technique.

In addition, when applying the kernel matching technique, farmers who used agricultural information saw an increase of 180 kg in productivity relative to their nonuser counterparts. The radius matching algorithm further supported these findings, revealing that farmers who employed agricultural information achieved an increase of about 209 kg in wheat productivity compared to nonusers. Similarly, the stratification matching algorithm indicated a productivity increase of 175 kg for those utilizing agricultural information.

Overall, all matching algorithms used in the analysis demonstrated that farmers who adopted agricultural information had higher productivity levels than those who did not. On average, the increase in output across all methods was 196.75 kg. These results suggest that any of the three matching techniques like nearest neighbor, kernel, or radius matching can reliably assess the impact of agricultural information utilization on wheat productivity in the East Gojjam Zone of Amhara, Ethiopia. This finding is consistent with the research by [42], who reported similar outcomes, and is corroborated by [60], who demonstrated that various matching algorithms could effectively quantify the effects of agricultural information on sorghum-producing farmers in Kenya.

## 5. Conclusion and Recommendations

The study demonstrates a statistically significant difference in wheat productivity between households that utilize agricultural information and those that do not, with the disparity largely explained by farmers' mobility, access to extension services, availability of farm inputs, and proximity to market centers. The application of multiple matching algorithms including nearest neighbor, kernel, radius, and stratification matching confirms the robustness of the findings, showing that farmers who effectively use agricultural information achieve an average increase of 196.75 kg in wheat production. This underscores that agricultural information is not merely an additional resource but a decisive factor in enhancing productivity, food security, and economic stability in East Gojjam Zone. Evidence from FGDs revealed that farmers with better access to extension services, media, and inputs consistently reported higher productivity, while those in remote areas highlighted mobility constraints, long distances to markets, and poor infrastructure as major barriers to information utilization. Similarly, KIIs with extension workers and district agricultural experts stressed that weak rural infrastructure, limited digital literacy, and inconsistent information delivery reduce the effectiveness of agricultural services, yet they confirmed that even basic interventions such as mobile-based advisory systems, farmer training, and improved road networks can significantly enhance productivity. Based on these insights, the study recommends that policymakers integrate agricultural information services into local development plans and strengthen extension systems through increased budget allocation, recruitment of trained personnel, and the adoption of mobile platforms for real-time support. District-level agricultural offices should implement training programs focused on digital literacy and the practical application of information while ensuring that farmers have timely access to quality inputs. Furthermore, partnerships with media outlets should be formalized to guarantee regular dissemination of localized agricultural content, particularly targeting remote and underserved areas. By implementing these measures, policymakers and development actors can create an enabling environment where smallholder farmers not only access agricultural information but also apply it effectively, thereby improving productivity, strengthening livelihoods, and advancing food security in the study area.

### 5.1. Limitations of the Study

This study has several limitations that should be acknowledged. First, it uses cross-sectional data, which limits the ability to capture changes over time or establish strong causal relationships. Second, although PSM controls for observable characteristics, it cannot account for unobserved factors such as farmers' motivation, risk tolerance, or access to informal networks, which may influence both information use and productivity. Third, the study focuses exclusively on wheat farmers in the East Gojjam Zone, which may limit the generalizability of the findings to other crops, regions, or farming contexts. Lastly, reliance on self-reported data introduces the risk of recall bias or social desirability bias, which may affect the accuracy of responses.

### 5.2. Suggestions for Future Research

Future research should consider longitudinal or panel data to better assess the causal impact of agricultural information utilization over time. Expanding the study to other regions and crop types would improve generalizability. Additionally, mixed methods research that integrates qualitative approaches could provide deeper insights into the behavioral, institutional, and technological factors influencing information uptake. Exploring the role of gender, youth, and digital literacy in information access and use would also add value to policy design.

## Figures and Tables

**Table 1:** Descriptive summary of selected variables used in estimations.

**Variables**	**Agricultural information**		**t** − **stat**/**x**^2^** test** (**p value)**
	**Users (** **n** = 168**)**	**Nonusers (** **n** = 235**)**	
Age (years)	42.76 (12.29)	41.45 (11.87)	−1.07 (0.86)
Sex (1 = male)	36.23	50.62	0.0008 (0.98)
Education (1 = above read and write)	24.56	39.70	5.44 (0.142)
Family size (number)	5.64 (0.17)	5.32 (0.14)	−1.47 (0.93)
Farm experience (years)	21.97 (0.97)	20.54 (0.77)	−1.16 (0.87)
Farm size (hectare)	1.89 (0.071)	1.83 (0.06)	−0.64 (0.74)
TLU (number)	5.45 (0.18)	5.379 (0.23)	0.24 (0.59)
Mobility of respondents (yes = 1)	8.68	20.10	−8.88 (0.003^∗∗∗^)
Social membership (yes = 1)	29.28	39	0.53 (0.466)
Access to extension services (yes = 1)	46.90	40.20	22.32 (0.000^∗∗∗^)
Utilization of printed outlets (1 = yes)	6.70	8.93	7.34 (0.007^∗∗∗^)
Access to farm inputs (1 = yes)	11.17	16.38	19.90 (0.000^∗∗∗^)
Access to credit (1 = yes)	24.81	31.51	1.19 (0.274)
Distance to market center in minutes	55.92 (3.11)	61.78 (2.98)	1.33 (0.092^∗^)
Distance from home to DC (minutes)	30.92 (1.40)	27.68 (1.16)	−1.78 (0.9626)
Access to training (1 = yes)	22	27.30	1.49 (0.222)
Use of electronic media (1 = yes)	25	24	13.91 (0.000^∗∗∗^)

*Note: T*-test and chi-square test used for continuous and categorical variables, respectively. Source: Filed data, 2023.

Statistical significant at ⁣^∗∗∗^*p* ≤ 0.01, and ⁣^∗^*p* < 0.10.

**Table 2:** Landholding size of respondents.

**Land size (ha)**	**Frequency**	**Percent**
Below 1 hectare	64	15.88
1–2 hectare	223	55.33
Above 3 hectares	116	28.78
Total	403	100

*Note:* Source: Filed data, 2023.

**Table 3:** Estimation of logit model on factors influencing information utilization on wheat productivity.

**Variables**	**Coefficient**	**Std. Err.**	**Z**
Age	0.001289	0.0145124	0.09
Gender	−0.1401133	0.2145252	−0.65
Educational status	−0.015539	0.0906802	−0.17
Total family size	0.0514032	0.0406596	1.26
Farm experience	0.0016008	0.0139777	0.11
Total farm size	0.0343571	0.0942778	−0.36
TLU	0.0279109	0.0274009	−1.02
Mobility of respondents	0.5345461	0.1601027	−3.34^∗∗∗^
Social membership	0.1417873	0.1557955	0.91
Extension services	0.8253299	0.2618249	3.15^∗∗∗^
Access to printed outlets	0.3714907	0.204125	1.82^∗^
Access to farm inputs	0.5453555	0.1567988	3.48^∗∗∗^
Access to credit	0.0561602	0.1445579	0.39
Distance home to market center	0.0035943	0.0017275	−2.08^∗∗^
Distance to development center	0.0048699	0.0039621	1.23
Access to training	−0.0247219	0.1485143	−0.17
Electronic media exposure	0.4945052	0.1564359	3.16^∗∗∗^
Constant	−1.320619	0.5811798	−2.27^∗∗^
Log likelihood	−236.59098		
Number of observations	403		
LR chi^2^ (17)	74.30		
Prob > chi^2^	0.0000		
*Pseudo* − *R*^2^	0.1357		

*Note:* Standard errors in parentheses. Source: Filed data, 2023.

⁣^∗∗∗^*p* ≤ 0.01, ⁣^∗∗^*p* ≤ 0.05, and ⁣^∗^*p* < 0.10.

**Table 4:** Balancing results from radius caliber matching.

**Variables**	**Unmatched (matched)**	**Mean**		**%bias**	**%red. bias**	**t** **-test**	**t** ** -test ** **p** > |**t**|
		**Treated**	**Control**				
Age	U (M)	42.76 (42.82)	41.46 (43.80)	10.8 (−8.2)	*M* = 24.5	1.07 (−0.76)	0.283 (0.446)
Gender	U (M)	0.87 (875)	0.868 (869)	0.3 (1.8)	*M* = 545	0.03 (0.17)	0.978 (0.868)
Educational status	U (M)	1.97 (1.962)	2.055 (1.847)	−9.3 (12.6)	*M* = −35	−0.93 (1.17)	0.353 (0.242)
Total family size	U (M)	5.64 (5.62)	5.32 (5.67)	14.7 (−2.1)	*M* = 85.8	1.47 (−0.19)	0.143 (0.846)
Farm experiences	U (M)	21.97 (22.05)	20.54 (22.71)	11.7 (−5.4)	*M* = 53.6	1.16 (−0.48)	0.247 (0.631)
Farm size	U (M)	1.89 (1.93)	1.83 (1.938)	6.5 (−1.2)	*M* = 81.0	0.64 (−0.11)	0.521 (0.913)
TLU	U (M)	5.45 (5.52)	5.38 (5.245)	2.5 (9.2)	*M* = −266	0.24 (0.98)	0.809 (0.327)
Mobility	U (M)	0.208 (0.217)	0.344 (0.258)	−30.8 (−9)	*M* = 70.4	3.01 (0.85)	0.003^∗∗∗^ (0.4)
Social membership	U (M)	0.702 (0.695)	0.668 (0.655)	7.4 (8.7)	*M* = 17.7	0.73 (0.73)	0.467 (0.467)
Extension services	U (M)	0.964 (0.962)	0.804 (0.98)	51.5 (5.0)	*M* = 90.3	4.85 (0.82)	0.000^∗∗∗^ (0.4)
Printed media access	U (M)	0.214 (0.186)	0.115 (0.202)	27.0 (4.2)	*M* = 84.4	2.73 (0.35)	0.007^∗∗∗^ (07)
Access to farm inputs	U (M)	0.191 (0.367)	0.191 (0.391)	45.3 (5.6)	*M* = 87.7	4.56 (0.46)	0.000^∗∗∗^ (0.6)
Credit access	U (M)	0.595 (0.608)	0.540 (0.630)	11.1 (4.4)	*M* = 60.3	1.09 (0.40)	0.275 (0.689)
Distance to market	U (M)	55.92 (56.42)	61.78 (57.64)	13.6 (−2.8)	*M* = 79.3	1.33 (−0.26)	0.184 (0.792)
Distance to development center	U (M)	30.92 (30.99)	27.68 (32.87)	18.0 (−10)	*M* = 42.1	1.79 (−0.83)	0.075^∗^ (0.41)
Access to training	U (M)	0.529 (0.534)	0.468 (0.543)	12.3 (−1.9)	*M* = 84.9	1.22 (−0.17)	0.223 (0.867)
Access to electronic media	U (M)	0.60 (0.583)	0.412 (0.534)	38.3 (10.1)	*M* = 73.6	3.79 (0.90)	0.000^∗∗∗^ (0.4)
Sample	Ps *R*^2^	LR chi^2^	*p* > *chi*^2^	MeanBias	MedBias	*B*	*R*
Unmatched	0.136 13	74.30	0.000	18.3	12.3	91.6^∗^	0.65
Matched	0.017	7.38	0.978	6.0	5.4	30.4^∗^	0.96

*Note:* Standard errors in parentheses. Source: Filed data, 2023.

⁣^∗∗∗^*p* ≤ 0.01, and ⁣^∗^*p* < 0.10.

**Table 5:** ATT of agricultural information utilization on wheat productivity.

**Matching algorithm**	**Participants**	**Nonparticipants**	**ATT**	**Std. Err.**	**t** ** value**
NNM	168	95	223	90	2.48^∗∗^
Kernel	168	230	180	85	2.12^∗∗^
Radius	166	230	209	92.5	2.26^∗∗^
Stratification	168	230	175	80	2.19^∗∗^

*Note:* Source: Filed data, 2023.

⁣^∗∗^*p* ≤ 0.05.

## Data Availability

The data that support the findings of this study are available from the corresponding author upon reasonable request.
